# Assessing the Treatment of Potential Effect Modifiers Informing World Health Organisation Guidelines for Environmental Noise

**DOI:** 10.3390/ijerph17010315

**Published:** 2020-01-02

**Authors:** Owen Douglas, Enda Murphy

**Affiliations:** School of Architecture, Planning and Environmental Policy, University College Dublin, Dublin 4, Ireland; enda.murphy@ucd.ie

**Keywords:** methodology, systematic reviews, confounders, mediators, moderators, effect modification, assessment, health, wellbeing, outcomes

## Abstract

Methodologies employed in the production of systematic reviews used to inform policy must be robust. In formulating the recent World Health Organisation (WHO) Environmental Noise Guidelines for the European Region, seven systematic reviews of evidence were commissioned to assess the relationship between environmental noise exposure and a range of health outcomes, six of which were nonauditory. Within the methodological guidance document devised for these reviews, inclusion and exclusion criteria for individual studies and existing reviews were applied in accordance with the Population-Exposure-Comparator-Outcome-Study (PECOS) framework for the evaluation of evidence. Specific criteria were defined for “populations” and source-specific “exposure”, but no criteria were defined for the treatment of potential “effect modifiers”. Furthermore, no criteria were set for the treatment of combined exposures. Employing a custom-designed assessment matrix, we assess the treatment of potential effect modifiers in the formulation of the aforementioned systematic reviews, all published in a Special Issue of the International Journal of Environmental Research and Public Health (IJERPH), titled “WHO Noise and Health Evidence Reviews”. We identify substantial methodological variation in their treatment and propose the differentiation of “moderators” and “mediators” from “confounders” as the basis for criteria development—including combined exposures—for future systematic reviews.

## 1. Introduction

### 1.1. Research Context

The World Health Organisation (WHO) recently published the Environmental Noise Guidelines for the European Region [1]. These guidelines are based on an assessment of evidence resulting from systematic literature reviews pertaining to associations between environmental noise exposure and cardiovascular and metabolic effects [2]; annoyance [3]; effects on sleep [4]; cognition [5]; hearing impairment and tinnitus [6]; adverse birth outcomes [7]; and quality of life, mental health and wellbeing [8]. Considered applicable in other regions and suitable for a global audience, the guidelines provide recommended noise limits for road, rail and aircraft using the L_den_ and L_night_ indicators for exposure at the most exposed building façade outdoors. L_den_ averages the noise out over a twelve hour day, a four hour evening, and an eight hour night, with 5 and 10 decibels added to the evening and night figures, respectively, to account for generally lower background levels at those times. L_night_ averages the noise over the 8 h night period (23:00 to 07:00).

While the new guidelines must be welcomed, a number of limitations relating to the systematic reviews which informed their development can be noted, not least the absence of consistent treatment of potential effect modifiers beyond a loose assessment of confounders. This is consequent on the absence of a definition of what constitutes a “confounder” within the methodology, or a standardised procedure for the treatment of potential effect modifiers [9]. The decision not to recommend inclusion or exclusion criteria for “confounders” was based on the assertion that other risk factors may confound the relationship between exposure to noise and a health outcome. The role of effect modifiers and combined exposures in potentially modifying associations between environmental noise exposure and population health outcomes is, therefore, not considered. Despite this, the methodology required assessment of “bias due to confounding”, but did not provide a standardised assessment method. Furthermore, the guidelines did not require consideration of combined exposure to different environmental stressors—for example, noise and vibration or noise and air pollution. In the document, however, the WHO explicitly acknowledges the need to develop comprehensive models to quantify the effects of multiple exposures on human health. Taken together, it can be argued that these issues greatly weaken the methodological strength of the informing systematic reviews and indicate the need for continued work to further update the guidelines. 

Given these identified shortcomings, the intention of this paper is to revisit the aforementioned systematic reviews and critically evaluate the treatment of identified “confounders” included in each review. First, we provide an overview and critique of the methodological guidance document as regards the treatment of potential effect modifiers. Based on this critique, we propose a categorisation procedure to systematically differentiate potential effect moderators and mediators from extraneous confounders. We then employ a standardised assessment matrix to assess each review according to their treatment of extraneous variables that potentially modify noise–health associations, including combined exposures. In doing this, we propose an assessment framework and demonstrate the need for a more refined methodology for the identification and treatment of potential effect modifiers. Such an approach permits the adoption of standardised inclusion and exclusion criteria and allows for more robust assessment of potential bias resulting from potentially modifying factors, including combined exposures, in future systematic reviews.

### 1.2. Methodological Guidance for WHO Systematic Reviews

As set out in the methodology document for systematic reviews for the WHO guidelines [9], the stated objectives of the evidence reviews were to assess the strength of association between exposure to environmental noise and incidence or prevalence of adverse health effects, and where possible, to quantify the risk of these health effects with an incremental increase in noise exposure. In pursuance of these objectives, the main research question was: “In the general population exposed to environmental noise, what is the exposure–response relationship between exposure to environmental noise (reported as various noise indicators) and the proportion of people with a validated measure of health outcome…, when adjusted for main confounders?”

In accordance with the Population-Exposure-Comparator-Outcome-Study (PECOS) framework for the evaluation of evidence, criteria were set for types of study to be considered, types of study participant, types of exposure measurement, and types of outcome measure, but no inclusion or exclusion criteria were set for the treatment of extraneous effect modifiers considered (or not considered) in studies under review. However, for each study, it was required that the “possible confounders taken into account” were recorded.

The grading of recommendations assessment, development and evaluation (GRADE) approach was adapted to assess the overall quality of evidence [10], with a view to “systematically and transparently” assessing the quality of the body of evidence for each outcome grouping based on specific factors—one of which was “plausible confounding, which would reduce a demonstrated effect”. The GRADE adaptations for the assessment of the quality of the evidence for health effects required that the assessment for a particular outcome would start as being high quality, where “all plausible residual confounding” is controlled for. According to the adapted methodology, individual studies should meet the inclusion and exclusion criteria set out in Annex 2 of the methodology document [9] in order to be included in the evidence reviews. The methodology states that criteria can be adjusted if needed for each review, but detailed justification should be given. Despite the lack of guidance for the identification and assessment of potential effect modifiers, the methodology required that among other factors, the quality and risk of confounding bias was assessed for individual studies included in the reviews. The template for such assessment is included in Annex 3 of the methodology document [9]. The template clearly directs that no inclusion or exclusion criteria should be applied for confounding, and no means of determining “all plausible residual confounding” are set out. Furthermore, it requires the subjective assessment of the type and number of confounders that should be incorporated for a valid assessment of associations between each noise source and the outcome under investigation. That is, a predetermined range of “important” confounders should be set by review teams, and the risk of bias judged based on the number of such confounders included in each study. These issues pose serious challenges for the consistent undertaking of systematic reviews according to this methodology, since no instruction is provided as to how to identify, categorise or assess the treatment of confounders or potentially wider modifying variables. That is, individual review teams would have to devise methods for counting and assessing confounders in individual studies (if at all), resulting in an inconsistent approach in the assessment of individual studies. This is a highly subjective procedure that is unlikely to be undertaken in a consistent manner by multiple review teams and for multiple health and wellbeing outcomes. 

### 1.3. Moderators, Mediators and Confounders

In analysing the relationship between environmental noise exposure and wide-ranging health outcomes, studies have increasingly attempted to control and account for the influence or potential influence of wide ranging variables. Such variables may, either independently or in combination, explain or partially explain a given association or nonassociation (e.g., gender, duration of exposure, sensitivity of persons exposed, etc.). The potential effect of such variables was recognised in some of the systematic reviews, being referred to as mediators (i.e., air pollution and long-term exposure) or listed in supplementary tables as “confounders”. However, limited assessment or categorisation was undertaken beyond noting their consideration in individual studies, despite the potential importance of such variables in assessing the validity of an identified association or nonassociation. Instead of being treated in a homogeneous manner and referred to as “confounders” as directed in the methodological guidance document [9], such influencing variables or potential effect modifiers can in fact be further categorised as moderating, mediating or confounding variables (or moderators, mediators and confounders), which need not be mutually exclusive. 

Moderators are interactions that change the size or direction (or both) of the effect of the exposure on an outcome. For environmental noise, such interactions might include the influence of building type (e.g., apartment, house, etc.), composition of rooms or internal layout, quantum of open space, noise sensitivity of individual subjects or respondents, pre-existing medical conditions, proximity to a given noise source or a combination of sources, fluctuating traffic flow, or the noise level from a given source at the point of measurement compared to other sources at the same noise level. 

Mediators lie on the causal pathway between exposure and outcome. In order to account for the potential mediating effects of intervening variables, recent studies have variously explored the influence of length of habitation, night work and exposure to air pollution in exploring the association between wide-ranging health and wellbeing outcomes and exposure to environmental noise. While potentially on the causal pathway, these mediators may themselves be related to other factors. For example, long-term habitation may be related to an inability of subjects or participants to purchase or rent a higher quality, more insulated dwelling [11,12]. As such, it may be the effect of confounding socioeconomic variables that better explains the relationship. 

Confounding variables are heterogeneous characteristics that jointly determine particular health outcomes and the level of exposure to environmental noise. These include sociodemographic and socioeconomic characteristics of the receiver, such as age, neighbourhood classification, gender, income category, marital status, educational attainment and employment status. They also include psychosocial factors, such as respondent perceptions. The status of exposed populations and individuals in terms of such characteristics has the potential to influence both their propensity to have a particular medical condition and their exposure to environmental noise. 

This approach to categorisation clearly demonstrates that the classification of all potential extraneous variables as “confounders” for the purpose of the WHO reviews potentially underplays their variety and potential role in modifying associations between environmental noise exposure and various health outcomes.

## 2. Materials and Methods 

With a view to assessing the WHO methodology as regards the identification and treatment of potential effect modifiers, we examine the six reviews for nonauditory health outcomes. The analysis is designed to identify potential pathways for methodological development for the future assessment of noise–health associations and subsequent policy guidance.

Each systematic review (and any supplementary document) was assessed to identify the treatment of extraneous variables, both in terms of identification and assessment. To facilitate this, we differentiated modifiers and mediators from extraneous confounders. We subsequently proposed and implemented a table matrix to undertake the assessment of each systematic review according to nine criteria. Informed by the WHO methodological document [9], the criteria are as follows: (1) consistent identification of specific confounders; (2) systematic scoring or ranking of risk of bias due to confounding; (3) application of inclusion and exclusion criteria for individual studies based on confounders; (4) consistent identification of specific moderators in individual studies; (5) consistent identification of specific mediators in individual studies; (6) Identification of any potential confounders; (7) identification of any potential moderators; (8) identification of any potential mediators; (9) identification of the potential role of combined exposures. 

In undertaking this assessment, we identify the strengths and weaknesses of the six systematic reviews as regards the identification and treatment of potential modifying variables in understanding the relationship between environmental noise exposure and nonauditory population health outcomes. In doing so, we assess the need for methodological development for assessing the relationship between environmental noise exposure and health outcomes, including the treatment of combined exposures. Each review is first considered in turn, before we present a combined assessment matrix to directly compare review approaches.

## 3. Results

### 3.1. Cardiovascular and Metabolic Effects

The systematic review on environmental noise and cardiovascular and metabolic effects was undertaken by van Kempen et al. [2]. Both the published article and a “full” review [13] were assessed for the purposes of this analysis. Table 1 comprises our assessment for these outcomes undertaken according to the methodology outlined.

For each selected study, the authors evaluated the risk of bias due to confounding (among other factors) based on a framework methodology developed by the WHO [14]. In adapting the GRADE approach, they evaluated whether the results of individual studies could be explained by possible confounding. The protocol they developed scores the risk of confounding bias as ‘low’, ‘high’ or ‘unclear’. A score of ‘low’ was applied where “all important confounders are taken into account either through matching, restriction or in the analysis”. A score of ‘high’ was applied where “only 1 or no confounder is taken into account, or subjects in exposed and unexposed groups differ for one or more important confounders and there is no adjustment in the analysis”. Finally, the designation of ‘unclear’ was applied where “less than all or >1 important confounder(s) taken into account, or insufficient information to decide on one of the above.”....Age and gender were identified as the “important” confounders for hypertension and blood pressure, while age, gender and smoking status were identified as the “important” confounders for Ischemic Heart Disease (IHD), stroke, type 2 diabetes, and obesity. That is, the risk of bias due to confounding was considered “low” if all “important” confounders were taken into account in individual studies, or “unclear” or “high” if only a portion or none were controlled for. Notably, the authors applied inclusion and exclusion criteria based on this assessment for confounding bias: “Since the extracted estimates had to be un-confounded by at least age and sex, we included estimates only from studies that were well-matched, adjusted or stratified for at least age and sex.” [13]. 

For risk of hypertension, for example, the authors surmised that most studies adjusted for “important” confounders [13], but less so for other cardiovascular and metabolic outcomes. This review provided measurable criteria for the treatment of confounders through the use of the scoring protocol. However, the range of confounders required to score a study as “low” in terms of confounding bias was limited to age, gender and smoking, at most. While comprising confounders according to the correct definition, this approach does not give any consideration to potentially wider modifying variables that may moderate or mediate relationships between environmental noise exposure and cardiovascular and metabolic health outcomes.

This review did make specific reference to the potential influence of combined exposures on cardiovascular health (particularly in reference to the review by Tétreault and Perron [15]). In particular, the authors focused on air pollution from transportation sources and the “mutually confounding” effect of noise or air pollution that may influence individual study results: “People living in a city or close to roads are exposed not only to traffic noise, but also to air pollution generated by traffic. Several studies indicate that exposure to air pollution may affect the cardiovascular system. Air pollution and noise from road traffic share the same source, so the effects could be attributed to both exposure types. This may give rise to confounding, where it is difficult to ascribe observed effects to a specific exposure, as well as to effect modification, where the two exposures interact in causing cardiovascular effects” [13]. In highlighting this relationship, the authors identify difficulty in ascribing observed effects to a specific exposure, but in doing so, flag the potential for the development of methodologies that can somehow unpick combined exposures.

### 3.2. Annoyance

Guski et al. [3] undertook a systematic review on environmental noise and annoyance. Having performed a literature search in 20 databases, studies that fulfilled selection criteria relating to study type, participants, exposure type, outcome measure, confounders and language were included in the formal meta-analysis. Our summary assessment for environmental noise exposure and annoyance is set out in Table 2.

For confounders, papers containing a potential second risk factor besides noise (e.g., vibrations in case of railway noise close to the tracks) were included in a re-analysis and received special remarks in the list of included papers. Author’s questionnaires were developed, which required the recording of noise metrics and confounders, if any, considered in statistical analyses included in each study [16]. For each source (road noise, rail noise, aircraft noise, and wind turbines), “confounding adjusted” was included as one of eight domains of assessment for the quality of evidence related to twenty metrics for exposure and annoyance (S1 to S20). In each case, the criterion for assessment was whether an effect was recorded in spite of confounding. For all sources, per the WHO guidance document, no adjustment or upgrade was deemed necessary in assessing and grading the studies included and did not influence the grading of the study quality. 

As set out in S13 of the supplementary document relating to the influence of codeterminants in aircraft noise studies, the authors stated that: “Individual (or personal) confounding or moderating within-study variables are not considered here, but it should be kept in mind that they are of great importance in explaining the variance of individual annoyance judgments—they often show correlations with individual annoyance judgments of the same strength as do noise levels.” In making this remark, the authors acknowledge the potential importance of confounding and modifying effects of extraneous variables in explaining variance of individual annoyance judgements in individual studies. Specifically, in S23 of the supplementary document, the authors discuss the influence of codeterminants in road traffic noise studies. They refer to their earlier statement in S13, and further emphasise the potential importance of wider modifying variables in understanding associations between road traffic noise and annoyance: “individual noise annoyance judgments of residents are to a large extent influenced by confounding or moderating personal variables (e.g., noise sensitivity, and coping capacity).” (16; pg.38 of 73). The authors explicitly state that they do not assess such within-study variables in their review. Instead however, they undertook an analysis of between-study codeterminants “which apply to many residents and should be taken into account when analysing noise annoyance from road traffic noise.” They state that “these factors also should be taken into account if results between different studies are to be compared.” 

The codeterminants they specifically considered included: (1) environmental conditions relating to the sound transmission between source and survey participants; (2) access to quietness; and (3) motorways vs. urban roads. In consideration of combined sources, the reviewers considered “how the (long-term) “total annoyance” judgment in situations involving at least two different noise sources is related to the long-term energetically summated noise levels of the combination of two noise sources”. 

The authors suggest that in order to counteract confounding, “it would have been desirable to perform meta-regressions involving several of the potential moderating factors as predictors in the same analysis. But this would require a greater number of studies.” (As a rule of thumb the ratio of the number of studies to the number of potentially moderating factors should be 10:1 or greater.) They further emphasise that the subgroup analyses reported in the review were explorative, but that they still have their value in that they point to the potential role of effect modifiers.

### 3.3. Effects on Sleep

The systematic review on environmental noise and sleep was undertaken by Basner and McGuire [4]. In undertaking the review, the authors synthesised 41 qualitative studies and 33 quantitative studies. Table 3 comprises our summary assessment for effects on sleep.

Similar to van Kempen et al. [13], who undertook the systematic review on environmental noise and cardiovascular and metabolic effects, Basner and McGuire [4] considered age and gender to be “important” confounders. However, in addition to these, they included socioeconomic status in defining confounders as variables associated with both the exposure and the outcome. Unlike van Kempen et al., they did not include smoking. In consideration of risk of bias and assessment of quality, in line with the WHO guidance document and the approach of van Kempen et al., they did not exclude studies based on whether or not they adjusted for confounding. They further justified this decision on the basis that “the use of these variables for adjustment was variable” across studies. 

For polysomnography-measured cortical awakenings for road, rail, and aircraft noise, the reviewers re-analysed four studies. For the analysis, each noise event was annotated with its maximum sound pressure level (L_AS,max_), the age and gender of the exposed subject, the day of the week (weekday or weekend), and time from sleep onset. Both unadjusted models and models adjusted for age, gender, weekday, and time from sleep onset. In the statistical analysis of the German Aerospace Center’s (DLR) STRAIN study [17] and the Franco-German cooperation in traffic research (DEUFRAKO) study [18], odds ratios adjusted for age and gender, day of the week (weekend or weekday), and time from sleep onset were also calculated. Interestingly, adjusting only marginally reduced the Odds Ratios (ORs), and all estimates were still significantly different from 1. Data for additional confounding variables were not available, and therefore were not included. 

### 3.4. Cognition

The systematic review on environmental noise and effects on cognition was undertaken by Clark and Paunovic [5]. This review focused solely on transportation sources (road traffic, aircraft, and train and railway noise). Quantitative nonexperimental studies published up to June 2015 were included. A total of 34 papers were identified, all of which were of child populations. Of these, 82% of the papers were of cross-sectional design, with fewer studies of longitudinal or intervention design. Table 4 comprises our assessment for this review.

The data extraction phase of this review included an evaluation of whether the study used matching or adjustment in the analysis for potential confounding factors, such as socioeconomic status, which has been shown to influence both noise exposure and cognitive performance. In applying the adapted GRADE methodology, evidence ratings could be upgraded or downgraded according to specific criteria, including the upgrade of evidence for an effect in spite of confounding working towards the null. As discussed previously, a clear weakness of the methodology is the lack of a downgrading option for the exclusion of a specific range of potentially modifying variables within studies, which may moderate, mediate or confound an identified association.

The review identified studies of noise effects pertaining to: reading and oral comprehension; cognitive impairment; short-term and long-term (episodic) memory; the association of noise exposure on children’s long-term or short-term memory; tests of attention; and executive function deficit. For each, an assessment of the risk of confounding bias was undertaken and rated as “low”, “moderate” or “high”. 

The review also identified a number of studies that took both noise and air pollution into account. The authors considered that future studies need to consider both exposures, as evidence is emerging that air pollution may impact on cognitive functioning across the life course. Indeed, exposure to air pollution during the prenatal period has been shown to impact on early childhood cognition (see for e.g. Sentís et al. [19]). Usefully, this review listed all “confounders” that were adjusted for in each study (or lack thereof). However, beyond listing, the treatment of such variables was not an important element in assessing the strength of evidence. While stating that “most of the studies took adequate account of sociodemographic confounding between noise exposure and cognitive performance”, no explanation as to what constitutes “adequate” was provided. Furthermore, recent studies have investigated cognitive impairment in adults [20,21,22,23], while this review was limited to effects on cognition in children.

### 3.5. Adverse Birth Outcomes 

Nieuwenhuijsen et al. [7] undertook a systematic review on environmental noise and adverse birth outcomes. They re-reviewed all of the papers on environmental noise and birth outcomes included in three “recent” systematic reviews and conducted a systematic search on noise and birth outcomes to update these reviews for the period June 2014 (end date of previous systematic review) to December 2016. They focused specifically on aircraft and road traffic noise and birth outcomes, including preterm birth, low birth weight, being small for gestational age and congenital malformations. They recommended that high quality studies are required to establish such associations and that future studies should apply robust exposure assessment methods (e.g., modelled or measured noise levels at bedroom façade); disentangle associations for different sources of noise, as well as daytime and night-time noise; evaluate the impacts of individual noise events; and most importantly for the present assessment, control the analyses for confounding factors, such as socioeconomic status, lifestyle factors, and other environmental factors, especially air pollution. Our summary assessment for this review is set out in Table 5.

Per the WHO criteria, no inclusion or exclusion criteria were applied for confounding factors in this review. That is, studies that did not control for potentially modifying variables were included, in addition to those which took possible confounders into account. For assessment of confounding, a score of 0 was applied when no confounding factors were considered, a score of 1 was applied when confounding factors were considered but some key confounders omitted. Finally, a score of 3 was applied when the authors deemed that careful consideration of confounders had been applied. Based on this scale, the maximum total score can be 14. 

Studies with a score of ≥10 were assessed as at low risk of bias, studies with a score from 6 to 9 were assessed as at unclear risk of bias, and studies with a score ≤5 were assessed as at high risk of bias. According to the authors, the GRADE principles were applied to the systematic review “in a reproducible and appropriate way for judgments about quality of evidence.” No studies were excluded from the evaluation. In undertaking the review, the authors compiled a table (Table 2 in published review) that summarised epidemiological studies on environmental aircraft noise exposure and birth outcomes according to multiple criteria including confounding factors. The specific range of “confounding” factors considered in each study (where relevant) was set out. Overall, however, the authors argued that many of the studies have serious limitations, such as not properly addressing confounding. They recommended that special attention should be paid to the exposure assessment and potential confounders, especially socioeconomic status and air pollution. Furthermore, they recommended that exposure assessment should not only include modelled data, but also measurements and noise perception, and should take into account behaviour, timing of exposure and building characteristics, such as acoustic properties (e.g., double-glazed windows, noise insulation, etc.) and bedroom orientation (towards or away from road, floor, etc.). 

The authors recognised that it is important to obtain information on potentially wider modifying variables, including other environmental data, such as air pollution, which may occur often simultaneously in the case of traffic noise and can affect pregnancy outcomes. They suggested that the accuracy level of assessment of confounders including air pollution should be at the same level of accuracy as the noise assessment to be able to make sensible comparisons of risk estimates. Furthermore, they assert that work is needed on the mechanisms explaining the possible relationships. Thus, the authors identify need for further studies with robust exposure assessment, including other potentially modifying variables, such as socioeconomic status and air pollution, and evaluating the role of noise sensitivity. In so doing, they clearly assert the need for deeper structured analysis.

### 3.6. Quality of Life, Mental Health and Wellbeing

Clark and Paunovic [8] undertook the systematic review on environmental noise and quality of life, wellbeing and mental health (these were the same authors who undertook the review on environmental noise and cognition). The review included quantitative studies of road traffic noise, aircraft noise, railway noise, and wind-turbine noise on children and adults in home and school environments published between January 2005 and October 2015. A total of 29 papers were identified, 90% of which were of cross-sectional design. Outcomes included depression and anxiety, medication use and childhood emotional problems. The quality of the evidence across the studies for each individual noise source was assessed using an adaptation of the GRADE methodology. Table 6 comprises our assessment for the review of quality of life, mental health and wellbeing.

In terms of confounding, the evaluation concluded that the majority of the studies were “adequate” in terms of taking sociodemographic confounding between noise exposure and mental health and wellbeing into account. However, as in the systematic review of environmental noise exposure and cognition undertaken by the same authors [5], no explanation as to what constitutes “adequate consideration” was provided.

This review identified studies of associations of environmental noise on self-reported quality of life; effects on medication intake for treatment of anxiety and depression; self-reported depression, anxiety and psychological symptoms; interview assessments of depression and anxiety disorders (often referred to as “common mental disorders” in the literature); emotional and conduct disorders in children, and hyperactivity in children. For each outcome, an assessment of adjustment for confounders was undertaken. As in their review pertaining to cognition, the authors categorised adjustment for confounding as “poor”, “fair” or “good”, and assessed the risk of bias due to confounding as “low”, “moderate” or “high”, however no criteria for such categorisations were set out. Furthermore, most of the studies did not take into account an individual’s history of mental ill-health, their ability to cope, their annoyance responses or their appraisal of the noise. These may be important codeterminants in the association and current studies may be over-simplifying the relationship between environmental noise and mental health and wellbeing. 

### 3.7. Combined Assessment Matrix 

Based on the individual assessments, a summary assessment matrix was developed to compare the approaches of each review according to the nine assessment criteria was compiled. This involved the combination of findings from the preceding assessments. A traffic light system was employed to visually specify whether individual studies met each of the nine criteria as set out, with green shading indicating “yes” and red shading indicating “no” (see Table 7). 

## 4. Discussion

From the outset of the present review, it was clear that weaknesses in the methodological guidance document resulted in the adoption of different approaches for each criterion by individual review teams. According to the matrix as set out in Table 7, the review pertaining to cardiovascular and metabolic effects by van Kempen et al. was the most comprehensive in matching the assessment criteria (6 of 9), while the review pertaining to quality of life, mental health and wellbeing by Clark and Paunovic (2018 b) matched the fewest criteria (2 of 9). No review matched all nine criteria. 

All six reviews for nonauditory health outcomes identified the potential role of confounders in modifying associations. However, only two reviews consistently identified specific confounders for specific health outcomes (cardiovascular and metabolic effects and effects on sleep). Age and gender were identified for all outcomes included in these two reviews, while smoking and socioeconomic status were identified for a limited range of outcomes. Two reviews systematically scored the risk of bias due to confounding factors (cardiovascular and metabolic effects and adverse birth outcomes), but applied different approaches based on the template for assessment of risk of confounding bias. One study employed inclusion and exclusion criteria for estimates from individual studies based on confounding (annoyance), while the remainder followed the guidance document in not applying such criteria for study selection. While not using the term itself, only one review identified a specific range of moderators in assessing studies (annoyance), while four more identified potential moderators without considering them systematically (all reviews except cardiovascular and metabolic effects). It would seem that this approach is at least in part a result of the wide definition of “confounder” employed by the authors of this review. Since the methodology document only referred to confounding, it is unsurprising that wider ranging potential effect modifiers were not considered systematically in most reviews. Indeed, potential effect moderators were identified and discussed in five of the reviews (all reviews except effects on sleep). Regarding mediators, air pollution was identified in four of the reviews as a matter for consideration (cardiovascular and metabolic effects; annoyance; cognition in children; adverse birth outcomes), but in line with the methodology document, such factors were not systematically assessed. However, these same four reviews clearly identified the need to consider the role of combined exposures (namely air pollution and noise) in understanding negative health effects.

This assessment indicates the need for a more consistent methodology for assessing the relationship between environmental noise exposure and health outcomes, including the treatment of combined exposures, particularly in terms of the treatment of potentially modifying variables. In the ideal review, all nine criteria should achieve a “yes” in the combined assessment matrix (Table 7) and be shaded green. That is, all 54 cells (100%) should achieve a “yes”, where only 25 (46.3%) in the current assessment attain this designation. This clearly highlights the need for more comprehensive and consistent methodological approaches for the assessment of evidence to inform guidance. A starting point for methodological enhancement would be to bring together the successful elements of each review and create specific and well-defined categorisations for “moderators”, “mediators” and “confounders”.

## 5. Conclusions

Owing to the growing body of evidence that exposure to noise from transportation sources increases the risk for wide-ranging health outcomes, the World Health Organisation (WHO) has acted to provide policy guidance, which aims to protect human populations from the adverse effects of environmental noise. In publishing the Environmental Noise Guidelines for the European Region (2018) and setting recommended exposure levels and limit values for noise exposure from multiple sources, the WHO has taken progressive measures towards protecting populations from the noise pollution problem. However, the guidelines are not without their shortcomings. Of particular importance to this study is that the systematic reviews that informed their formulation—while methodologically robust for most aspects—were not consistent in their consideration of potential effect modifiers in individual studies. This is consequent of a number of weaknesses associated with the methodology for systematic reviews, which was limited in its definition of potential effect modifiers and its consideration of the importance of such effects, including the role of combined exposures.

The absence of inclusion and exclusion or detailed assessment criteria for potential effect modifiers from the WHO methodological document inevitably resulted in their inconsistent treatment in the individual systematic reviews. As a result, the strength of the methodology and the findings and recommendations of the subsequent reviews can be justifiably critiqued. Indeed, the recommended exposure levels and updated limit values for exposure to noise from individual sources were set by the WHO based on the findings of these reviews. As such, it is questionable whether the guideline limit values provided would be the same had a more consistent methodological approach been adopted by review teams.

This study is useful because by means of a purpose-designed assessment matrix, the treatment of potential effect modifiers in the WHO reviews of noise exposure and nonauditory health effects are assessed in a consistent manner. This allows for cross-comparison, both in terms of approach and for identifying strengths and weaknesses in each review according to the nine criteria. In this way, it provides a basis for the enhancement of the methodology protocol, both in terms of clarity and scope. In doing so, we provide a solid justification for further studies and systematic reviews that (1) systematically and consistently consider the influence of moderating, mediating and confounding factors in noise–health associations; and (2) investigate the combined effects of multiple and varied noise sources and other environmental stressors on population health and wellbeing.

An implication of our findings is that comprehensive re-reviews of evidence will be required to take better account of the role of potential effect modifiers that have been otherwise excluded from, or whose potential effects have been understated, in the systematic reviews assessed here. Such reviews would act to test the assessment matrix proposed and implemented in this paper, as well as improve our understanding of the full extent of evidence for environmental noise exposure and population health outcomes. In particular, the systematic assessment of modifiers, mediators and confounders, in addition to consistent investigation of combined effects of multiple sources and other environmental stressors, should form the basis of methodological advancement for undertaking future reviews. In particular, methodological advancements for the assessment of the impact of combined sources, population and built environment characteristics require increased attention, both for threshold specification and for the abatement and mitigation of negative health impacts from environmental noise in heterogeneous populations.

## Figures and Tables

**Table 1:** Assessment for cardiovascular and metabolic effects.

Criteria	Assessment
Consistent identification of specific confounders	Yes: Age and gender for hypertension and blood pressure. Age, gender and smoking for Ischemic Heart Disease (IHD), stroke, type 2 diabetes, and obesity.
Systematic scoring or ranking of risk of bias due to confounding	Yes: High if only 1 or no confounder is taken into account, or subjects in exposed and unexposed groups differ for one or more important confounders and there is no adjustment in the analysis. Unclear if less than all or >1 important confounder(s) taken into account, or insufficient information to decide on one of the above.
Application of inclusion/exclusion criteria for individual studies based on confounders	Yes: Included estimates only from studies that were well matched, adjusted or stratified for at least age and sex
Consistent identification of specific moderators in individual studies	No
Consistent identification of specific mediators in individual studies	No
Identification of any potential confounders	Yes: As above
Identification of any potential moderators	No
Identification of any potential mediators	Yes: Air pollution
Identification of the potential role of combined exposures	Yes: Environmental noise and air pollution

**Table 2:** Assessment matrix for annoyance.

Criteria	Assessment
Consistent identification of specific confounders	No: Did not assess within-study variables
Systematic scoring or ranking of risk of bias due to confounding	No: In line with the World Health Organisation (WHO) guidance document
Application of inclusion/exclusion criteria for individual studies based on confounders	No
Consistent identification of specific moderators in individual studies	Yes: Environmental conditions relating to the sound transmission between source and survey participants; access to quietness; motorway vs. urban roads
Consistent identification of specific mediators in individual studies	No
Identification of any potential confounders	Yes: Acknowledged the importance of confounding factors, for example identified potential confounders associated with the HYENA* study (age range, two change airports, face-to-face-interviews, annoyance question related to daytime) as the likely explanatory factors for stronger correlations
Identification of any potential moderators	Yes: Authors acknowledged the importance of moderating (e.g., noise sensitivity and coping capacity)
Identification of any potential mediators	Yes: Air pollution and long-term exposure
Identification of the potential role of combined exposures	Yes: Papers containing a potential second risk factor besides noise were included and received special remarks in the list of included papers. The authors identified that in addition to the personal within-study factors, there are several codeterminants within- and between-study factors which should be taken into account when analysing noise annoyance from combined noise (e.g., situational factors such as distance to the noise source). The reviewers considered how the (long-term) “total annoyance” judgment in situations involving at least two different noise sources is related to the long-term energetically summated noise levels of the combination of two noise sources.

* HYpertension and Exposure to Noise near Airports.

**Table 3:** Assessment for effects on sleep.

Criteria	Assessment
Consistent identification of specific confounders	Yes: Age, gender and socioeconomic status considered “important” confounders. In the re-analysis conducted for this review, models adjusted for age, gender, weekday, and time from sleep onset.
Systematic scoring or ranking of risk of bias due to confounding	No: The use of these variables for adjustment was deemed to be variable across studies.
Application of inclusion/exclusion criteria for individual studies based on confounders	No
Consistent identification of specific moderators in individual studies	No
Consistent identification of specific mediators in individual studies	No
Identification of any potential confounders	Yes: As above, plus pre-existing health conditions and level of parental education (in the case of children)
Identification of any potential moderators	Yes: Pre-existing medical conditions, homogeneous study populations, duration of sleep, sensitivity and quiet façade.
Identification of any potential mediators	No
Identification of the potential role of combined exposures	No

**Table 4:** Assessment for effects on cognition.

Criteria	Assessment
Consistent identification of specific confounders	No: Authors purported that most of the studies included in this review took “adequate account” of sociodemographic confounding between noise exposure and cognitive performance. However, as noted by the authors, older studies from the 1970s and 1980s are considerably less likely to have taken even socioeconomic confounding into account.
Systematic scoring or ranking of risk of bias due to confounding	No: Authors concluded that studies made “good adjustment” for socioeconomic and other confounders and participants. However, no criteria were set out for what qualifies as “good adjustment” or what constitutes the full range of potential confounders.
Application of inclusion/exclusion criteria for individual studies based on confounders	No: Due to the omission of control for individual level socioeconomic confounding. However, downgrading was applied to some individual studies in terms of their quality assessment on the basis of confounding bias.
Consistent identification of specific moderators in individual studies	No
Consistent identification of specific mediators in individual studies	No
Identification of any potential confounders	Yes: Downgrades of quality specifically due to the rating of high risk of bias associated with residual socioeconomic confounding in longitudinal and intervention studies.
Identification of any potential moderators	Yes: Moderators such as building characteristics but referred to as “confounders”.
Identification of any potential mediators	Yes: Air pollution.
Identification of the potential role of combined exposures	Yes: Considered that future studies need to consider both noise pollution and air pollution exposure.

**Table 5:** Assessment for effects on birth outcomes.

Criteria	Assessment
Consistent identification of specific confounders	No: Due to poor control for confounding factors in existing studies, such as “socioeconomic status, lifestyle factors and other environmental factors, especially air pollution.”
Systematic scoring or ranking of risk of bias due to confounding factors	Yes: A score of 0 was applied when no confounding factors were considered, a score of 1 was applied when confounding factors were considered but some key confounders were omitted. Finally, a score of 3 was applied when the authors deemed that careful consideration of confounders had been applied. Studies with a score of >10 were assessed as at low risk of bias, studies with a score from 6 to 9 were assessed as at unclear risk of bias, and studies with a score <5 were assessed as at high risk of bias.
Application of inclusion/exclusion criteria for individual studies based on confounders	No: Per the WHO inclusion and exclusion criteria table, no inclusion or exclusion criteria were applied for confounding factors in this review.
Consistent identification of specific moderators in individual studies	No: Due to poor control for extraneous factors in existing studies.
Consistent identification of specific mediators in individual studies	No
Identification of any potential confounders	Yes: As above
Identification of any potential moderators	Yes: Moderators including as noise level from a given source, noise sensitivity, noise perception, individual behaviour, timing of exposure and building characteristics, such as its acoustic properties (e.g., double-glazed windows, noise insulation, etc.) and bedroom orientation (towards or away from road, floor, etc.). All referred to as “confounders”.
Identification of any potential mediators	Yes: Air pollution.
Identification of the potential role of combined exposures	Yes: Other environmental data such as air pollution, which may occur often simultaneously in the case of traffic noise and can affect pregnancy outcomes.

**Table 6:** Assessment for effects on quality of life, mental health and wellbeing.

Criteria	Assessment
Consistent identification of specific confounders	No: The evaluation concluded that the majority of the studies were “adequate” or “good” in terms of taking sociodemographic confounding between noise exposure and mental health/wellbeing into account. They noted that “residual confounding may remain”. No explanation as to what constitutes “adequate” or “good” control for confounding factors.
Systematic scoring or ranking of risk of bias due to confounding factors	No: The risk of bias in individual studies was generally judged to be “low”, with good control for socioeconomic confounding. No criteria set out for what qualifies as “low” risk or “good control” for socioeconomic confounding.
Application of inclusion/exclusion criteria for individual studies based on confounders	No
Consistent identification of specific moderators in individual studies	No
Consistent identification of specific mediators in individual studies	No
Identification of any potential confounders	Yes: As above
Identification of any potential moderators	Yes: Individual’s history of mental ill-health, their ability to cope, their annoyance responses or their appraisal of the noise.
Identification of any potential mediators	No
Identification of the potential role of combined exposures	No

**Table 7:** Combined assessment matrix.

Criteria	Assessment Matrix					
	Cardiovascular and Metabolic Effects, van Kempen et al. [2]	Annoyance, Guski et al. [3]	Effects on Sleep, Basner and McGuire [4]	Cognition (Children Only), Clark and Paunovic [5]	Adverse Birth Outcomes, Nieuwenhuijsen et al. [7]	Quality of Life, Mental Health and Wellbeing, Clark and Paunovic [8]
Consistent identification of specific confounders	Yes	No	Yes	No	No	No
Systematic scoring or ranking of risk of bias due to confounding	Yes	No	No	No	Yes	No
Application of inclusion/exclusion criteria for individual studies based on confounders	Yes	No	No	No	No	No
Consistent identification of specific moderators in individual studies	No	Yes	No	No	No	No
Consistent identification of specific mediators in individual studies	No	No	No	No	No	No
Identification of any potential confounders	Yes	Yes	Yes	Yes	Yes	Yes
Identification of any potential moderators	No	Yes	Yes	Yes	Yes	Yes
Identification of any potential mediators	Yes	Yes	No	Yes	Yes	No
Identification of the potential role of combined exposures	Yes	Yes	No	Yes	Yes	No
